# Prevalence and Associated Risk Factors of Bovine Fasciolosis in Injibara Town, North Western Ethiopia

**DOI:** 10.1016/j.vas.2026.100826

**Published:** 2026-08-22

**Authors:** Gashaw Molla Beza, Habtamu Addis, Melaku Yismaw

**Affiliations:** Department of Veterinary Laboratory Technology**,** Injibara University**,** Injibara**,** Ethiopia

**Keywords:** Bovine, Fasciolosis, Injibara town, Prevalence, Risk factor

## Abstract

Fasciolosis is a snail-borne parasitic disease caused by genus Fasciola liver flukes that leads to high morbidity and moderate to high mortality in cattle. A cross-sectional study was conducted from February to may 2026 to determine the prevalence and associated risk factors of bovine fasciolosis in Injibara town. A total of 384 cattle faecal samples were collected using simple random sampling techniques and subjected to the faecal sedimentation technique. The overall prevalence of fasciolosis was 122 (31.8%) [95%, CI=27.3-36.6]. Statistically significant variation (P < 0.05) was observed in the prevalence of fasciolosis among animals with different body conditions, ages and breeds. However, there was no statistically significant variation (P > 0.05) in the prevalence of fasciolosis based on sexes and origin of the animal. The present study revealed that infection of cattle by fasciolosis was attributed to the presence of favorable environment for the abundance of intermediate host and the parasite. The studies confirm the endemicity of fascioliasis at the study grazing lands. Appropriate strategies for the control of fascioliasis based on seasonal de-worming approach are suggested.

## 1 Introduction

Ethiopia is believed to have the largest livestock population in Africa and fifth in the world, with the estimated domestic animals are 61.5 million cattle, 33 million sheep, 38.9 million goats, 1.76 million camels and over 59.4 million poultry (CSA, 2022). Livestock production is a key component of the agricultural economy in developing countries, and it is a tool for socio-economic development, increased income, and improved rural life quality to contributing meat, milk, and eggs as vital protein sources (Sennuga *et al*., 2022; Pandey *et al*., 2026).

However, the country’s vast cattle potential is remained marginal due to low genetic quality of local breeds, prevailing diseases, poor nutrition, poor animal production systems, reproductive inefficiency, management constraint and a general lack of veterinary care (Assefa *et al*., 2026). Parasite infection, particularly common nematode infections, severely compromise livestock health while directly disrupting the domestic and global trade of cattle and food products (Strydom *et al*., 2023). Fascioliasis is a global zoonotic disease caused by two liver fluke species, *Fasciola hepatica* and *Fasciola gigantica*, which inhabit the liver and bile ducts of ruminants (Lan *et al*., 2024; Xu *et al*., 2025; Zulqarnain *et al*., 2025). The life cycle of both species is typical of digenetic trematodes characterized by indirect life cycles (Lalor *et al*., 2021). Animals with fasciolosis typically suffer from prolonged fever, hepatomegaly, eosinophilia, anorexia, weight loss, anaemia, liver damage, and even death (Regasa and Seboka, 2021).

Fascioliasis infected livers showed a number of pathological lesions, including bile duct hyperplasia, dilatation of lymphatic vessels in portal regions with extensive fibrosis, necrosis of the liver parenchyma, and Glisson’s capsule thickening with fibrosis (Hassan *et al*., 2025). Numerous techniques, including molecular approaches, parasitological examination, and serological investigation, have been used to diagnose fascioliasis (Thakur, 2024). Fasciolosis can cause huge economic losses due to decrease in milk and meat production, decreased feed conversion ratio, and cost of treatment (Strydom *et al*., 2023). Early detection of fasciolosis, treatment of animals with the best anthelmintic, strategic control of the intermediate host, effective use of grazing methods, and use of different antigens as a prophylactic treatment are the best strategies to control the disease (Rizwan *et al*., 2022).

In Ethiopia, *F. hepatica* covers over three-quarters of the country at altitudes above 1800 meters, while *F. gigantica* is localized to the humid western zone below 1200 meters, though their ranges can overlap in shared ecological zones (Japaro and Anebe, 2023). Ultimately, the prevalence of both parasites is determined by marshy habitats that support large populations of their intermediate snail hosts (Wamile *et al*., 2026). The prevalence of cattle fasciolosis by coprological examination in Ethiopia includes in Buno Bedelle Zone (23.7%) (Husen *et al*., 2017), Gurage Zone (49.3%) (Diilbato and Bekele, 2018), in and around Bahir Dar (20.1%) (Aragaw and Tilahun, 2019), Wolayta Sodo (23.7%) (Alemu, 2019), East Hararghe (30.5%) (Bilal and Musa, 2021), bale zone (48%) (Jilo *et al*., 2021), Arsi zone (37.5%) (Roba, 2022), West Hararghe (20.5%) (Shafi and Elemo, 2022), Toke Kutaye district (7.83%) (Wayessa *et al*., 2022), Wolayta Sodo (26.04%) (Kebede *et al*., 2023), Bahir Dar (49.21%) (Mesfin *et al*., 2026), and Hawassa Town (19.6%) (Dubie *et al*., 2026).

Despite cattle's vital agricultural role in Injibara town, there is currently no information available regarding the prevalence of bovine fasciolosis in the area. When left untreated, fasciolosis causes severe health complications, including unthriftiness, anemia, submandibular edema, and reduced milk production. Therefore, this study aims to determine the prevalence and major risk factors of bovine fasciolosis in Injibara town, providing the baseline data needed to formulate appropriate management measures.

## 2 Materials and methods

### 2.1 The Study Area

The study was conducted in and around Injibara town, Northwestern Ethiopia. It is the capital city of Awi Nationality Administrative zone located at 114 km form capital city of Amhara Regional state, Bahir Dar and 445 km from the capital city of Ethiopia Addis Ababa. The area lies between 10°50′16′′ and 11°58′50′′N latitude and from 36°50′20′′ to 37°02′10′′E longitude at an altitude ranging from 2,560 meter (8,400 ft) above sea level. The mean annual rainfall ranges from 1813 mm. The area is characterized by a high rainfall distribution. The mean annual temperature is 22.84°C and lies in the cold thermal zone of Ethiopia. The area is also categorized as moist dega zone as per traditional system and Temperate rainy climate (Mulugeta *et al*., 2017; NMA, 2021).

### 2.2 Study Design and Population

A cross-sectional study was conducted from February to May 2026. The study population recruited for this study was cattle having different sex, breed, age and body conditions, and origin kept under both semi-intensive and extensive management system living in Injibara town. Data on species, sex, age, origin, and body condition were organized by owner interview and physical inspection. Age evaluation relied on the dentition and was grouped into three categories, young (<1 year), adult (2-5 years), and old (>5 years), as defined by Pope and George (2008). Visual examination classified the body condition as being poor, medium, or good according to the standards of Nicholson and Butterworth (1986).

### 2.3 Sample size determination and sampling technique

The animals were selected by using simple random sampling method. To determine the sample size, an expected prevalence of 50% was taken into consideration since there was no research work on bovine fasciolosis in the area.

The desired sample size for the study was calculated using the formula given by Thursfield (2005) with 95% confidence interval and 5% absolute precision the sample size is calculated as follows:

n=1.96^2^ *P_exp_ (1-P_exp_) /d^2^

Where:

*n* = required sample size

*Pₑₓₚ* = expected prevalence (50%)

*d* = desired absolute precision (5%)

Hence the total number of animals that are going to be sampled is 384, from target population in the study area.

### 2.4 Laboratory Examination

A total of 384 fecal samples were collected during the entire period of the study directly from the rectum of selected animal using a gloved hand and place into a universal bottle containing 10% formalin. Samples were transported to Injibara University Veterinary Parasitology Laboratory as fresh as possible. Sedimentation technique was used to detect the presence or absence of fluke eggs in the fecal sample collected. A drop of methylene blue solution was added to the sediment where eggs of *Fasciola* species show yellowish color (Mathewos *et al*., 2023). During sampling information on sex, breed, body condition, origin and approximate age of individual animals were recorded. Samples that were not processed within 24 h were stored in a refrigerator at 4°C.

### 2.5 Data management and analysis

The collected data was coded and entered into Microsoft Excel for organization and then statistical analyses was performed using SPSS. Descriptive statistics was used to calculate prevalence while chi-square (χ2) test, univariate and multivariant logistic regression were used to compare the association of bovine fasciolosis with different risk factors. In all the cases, 95% confidence level and 0.05 absolute precision errors were considered. A p-value ≤ 0.05 was considered statistically significant.

### 2.5 Declarations

#### 2.5.1 Ethical Approval

All laboratory procedures and bovine management were compliant with internationally recognized guidelines regarding research animal handling and use. This study has an ethical clearance certificate (Ref. No. VET/LB/207.20/02/2026) obtained from the Injibara University Research Ethics Review Board prior to the initiation of any research activities. Fecal sample collection was non-invasive, and verbal informed consent was obtained from animal owners prior to sampling.

#### 2.5.2 Data Availability Statement

All data generated or analyzed during this study are included in this published article and its supplementary information files.

#### 2.5.3 Competing Interests

The authors declare that they have no competing interests. Funding Not Applicable Author.

#### 2.5.4 Funding

Funding None

## 3 Results

The study examined 384 fecal samples using the sedimentation technique and found an overall fasciolosis prevalence of 31.8% (122 infected animals) [95%, CI=27.3-36.6]. By sex, prevalence was higher in males than in females, this difference was not statistically significant (p > 0.05).

Bovine fasciolosis prevalence varied significantly by breed (P < 0.05), with a higher rate observed in crossbreeds than in local breeds. A statistically significant difference in fasciolosis prevalence was observed among the different age groups (P < 0.05). The infection rate was highest in old cattle, followed by adults, and lowest in young animals.

Analysis of body condition scores revealed a significant link to fasciolosis infection (P < 0.05). The prevalence decreased progressively from poor to medium and good body conditions. By geographic origin, the prevalence of bovine fasciolosis was in Entoto (32.3%), Kera (32.5%), Beata (29.8%), and Family (32.2%). Statistical analysis revealed no significant association between the origin of the animals and parasite prevalence (P > 0.05). All data summarized in Table 1.

**Table 1:** the prevalence of bovine fasciolosis with associated factors.

Risk factor	No. of examined cattle	No. of positive cattle	Prevalence (%)	χ2	P-value
Sex					
Male	216	75	34.7	1.984	0.159
Female	168	47	28.0		
Age					
Young	138	33	23.9	7.550	0.023
Adult	174	59	33.9		
Old	72	30	41.7		
Body condition					
Poor	39	29	74.4	37.939	0.000
Medium	69	23	33.3		
Good	276	70	25.4		
Breed					
Local	200	49	24.5	10.179	0.001
Cross	184	73	39.7		
Origin Entoto	99	32	32.3	0.202	0.9779
Kera	114	37	32.5		
Beata	84	25	29.8		
Family	87	28	32.2		

The association between bovine fasciolosis and several factors was evaluated using a univariate logistic regression model (Table 2). The analysis revealed that animal age, body condition score, and breed were significantly associated with the prevalence of fasciolosis infection (p < 0.05). Conversely, host sex (p = 0.160) and geographical origin (p > 0.05) did not show any statistically significant relationship with the occurrence of the disease. The highest prevalence of infection was recorded in older cattle (41.7%) and those with poor body condition scores (74.4%). Crossbreed cattle demonstrated a significantly higher risk of infection compared to local breeds, with crossbreeds being twice as likely to harbor the parasite [OR = 2.03, 95% CI = 1.31–3.14, p = 0.002].

**Table 2:** Univariate logistic regression analysis of risk factors.

Risk factor	No. of examined cattle	No. of positive cattle	Prevalence (%)	OR	95% CI	P-value
Sex						
Male	216	75	34.7	0.73 Ref	0.47-1.13	0.160
Female	168	47	28.0			
Age						
Young	138	33	23.9	Ref 0.718 0.44		0.25 0.008
Adult	174	59	33.9		0.41-1.26	
Old	72	30	41.7		0.24-0.81	
Body condition						
Poor	39	29	74.4	0.17 0.12 Ref	0.072-0.41	0.000 0.000
Medium	69	23	33.3		0.054-0.25	
Good	276	70	25.4			
Breed						
Local	200	49	24.5	Ref 2.03		0.002
Cross	184	73	39.7		1.31-3.14	
Origin Entoto	99	32	32.3	0.988	0.54-1.79	0.967
Kera	114	37	32.5	0.994	0.56-1.767	0.984
Family	87	28	32.2	0.88	0.48-1.62	0.686
Beata	84	25	29.8	Ref		

All risk factors that showed significant or borderline associations in the univariate analysis were further evaluated using a multivariate logistic regression model to control for confounding variables (Table 3). Age, body condition score, and breed remained robust, independent predictors of bovine fasciolosis.

**Table 3:** Multivariate logistic regression of bovine fasciolosis with associated factors.

Risk factor	No. of examined cattle	No. of positive cattle	Prevalence (%)	OR	95% CI	P-value
Age						
Young	138	33	23.9	Ref 0.459 0.82		0.522 0.021
Adult	174	59	33.9		0.24-0.89	
Old	72	30	41.7		0.45-1.51	
Body condition						
Poor	39	29	74.4	0.153 0.107 Ref	0.062-0.38	0.000 0.000
Medium	69	23	33.3		0.048-0.24	
Good	276	70	25.4			
Breed						
Local	200	49	24.5	Ref 2.04		0.003
Cross	184	73	39.7		1.27-3.25	

When holding other variables constant, animal age remained significantly associated with infection status (p = 0.021). Cattle categorized as adults showed a significantly reduced risk of infection compared to the younger reference cohort [OR = 0.459, 95% CI = 0.24–0.89]. Animal body condition was also highly predictive of infection (p < 0.001). Cattle with poor body condition [OR = 0.153, 95% CI = 0.06–0.38] and medium body condition [OR = 0.107, 95% CI = 0.05–0.24] displayed significant variations in infection odds relative to the good body condition cohort. Finally, host genetics remained a major determinant of disease susceptibility; crossbreed animals were twice as likely to test positive for fasciolosis compared to their local breed counterparts [OR = 2.04, 95% CI = 1.27–3.25, p = 0.003].

## 4 Discussions

Fasciolosis is a prevailing ruminant health predicament and causes substantial economic losses to the livestock commerce in Ethiopia. The current prevalence of bovine fasciolosis on coprological examination was 31.8%. The fecal sedimentation technique is a standard veterinary test for finding liver fluke and other trematode eggs. It often misses low-level, prepatent, or intermittent infections. This low sensitivity means true parasite rates in animals are usually higher than test results show (Carneiro *et al*., 2018). The present studies under the range of studies by Lan *et al*. (2024) and Wamile *et al*. (2026), which reported a prevalence of bovine fasciolosis internationaly at range of 12.02% to 96.67% while in Ethiopia from 11.5% to 87% (Nota and Dima, 2018), and aligns with Bilal and Musa (2021), Delgado *et al*. (2023), andRoka and Singh (2024).

This finding is relatively higher than in Egypt (9.77%) (El-Tahawy *et al*., 2017), Buno Bedelle Zone (23.7%) (Husen *et al*., 2017), Western Ethiopia (27.6%) (Dereje and Surra, 2018), Botswana (0.74%) (Mochankana and Robertson, 2018), Bahir Dar (20.1%) (Aragaw and Tilahun, 2019), South Sudan (19%) (Nigo *et al*., 2019), Wolayta Sodo (23.7%) (Alemu, 2019), Nigeria (18.3%) (Odeniran *et al*., 2020), Malaysia (21.6%) (Ahmad-Najib *et al*., 2021), Bedelle (19.27%) (Shafi, 2021), Thailand (20.3%) (Thanasuwan *et al*., 2021), Indonesia (16.50%) (Kurnianto *et al*., 2022), West Hararghe (20.5%) (Shafi and Elemo, 2022), Toke Kutaye district (7.83%) (Wayessa *et al*., 2022), Wolayta Sodo (26.04%) (Kebede *et al*., 2023), Southern Ethiopia (20.3%) (Mathewos *et al*., 2023), Rwanda (20%) (Tumusiime *et al*., 2023), and Hawassa Town (19.6%) (Dubie *et al*., 2026).

Higher prevalence was reported by Diilbato and Bekele (2018) in Gurage Zone (49.3%), Mohammed *et al*. (2018) in Eastern Shoa (54.2%), Khan *et al*. (2020) in Pakistan (42.8%), Diaz-Quevedo *et al*. (2021) in Amazonas Peru (90.13%), Jilo *et al*. (2021) in bale zone (48%), Kipyegen *et al*. (2022) in Kenya (46.0%), Roba (2022) in Arsi zone (37.5%), Frias *et al*. (2023) in Peruvian Amazon (45.6%), Muhammad *et al*. (2023) in Malaysia (45.8%), Geja and Aliyi, (2024) in Haromaya (47.9%), Nkurunziza *et al*. (2024) in Burundi (47.7%), Mesfin *et al*. (2026) in Bahir Dar (49.21%), and Vitouley *et al*. (2026) in Southwestern Burkina Faso (40%). This variation in the prevalence of bovine fasciolosis might be due to differences in environmental factors, management systems, and animal breeds, which contribute to differences in the ecology of the study areas, animal management systems, and the presence of nearby veterinary service (Xu *et al*., 2025).

The current study revealed that prevalence was higher in males than in females. Even if there were differences in prevalence, there was no significant difference (P > 0.05) between the two groups. Sex did not affect the prevalence of the disease, because the grazing pasture land or management system was the same and equally exposed to a similar contaminated pasture by both sex groups and traditionally animals are driven to pasture regardless of sex. It strongly aligns with a research, including studies by Dereje and Surra (2018), Nigo *et al*. (2019), Jilo *et al*. (2021), Kurnianto *et al*. (2022), Mathewos *et al*. (2023),Roka and Singh (2024), Dubie *et al*. (2026), Mesfin *et al*. (2026), and Vitouley *et al*. (2026). While this result contradicts the trends reported by Mohammed *et al*. (2018), Khan *et al*. (2020), Ahmad-Najib *et al*. (2021), Diaz-Quevedo *et al*. (2021), Frias *et al*. (2023), and Geja and Aliyi (2024).

Adult cattle serve as definitive hosts for the Fasciola parasite, with older animals exhibiting higher susceptibility due to prolonged exposure and acting as a continuous source of infection for younger livestock (Chongmobmi and Panda, 2018); (Mesfin *et al*., 2026). The investigation revealed a higher prevalence in old cattle compared to young and adult, aligning with other studies Mochankana and Robertson (2018), El-Tahawy *et al*. (2017), Nigo *et al*. (2019), Shafi (2021), Mathewos *et al*. (2023), and Geja and Aliyi (2024) regarding young animal’s low rate of infection than old animals. This finding is since bovine fasciolosis is a chronic disease, the higher age reflects a much longer period of exposure to infection, the maternal immunity acquired by young animals through colostrum. It disagrees with Mohammed *et al*. (2018), Dereje and Surra (2018), Khan *et al*. (2020), Jilo *et al*. (2021) andRoka and Singh (2024).

Our investigation revealed that the prevalence of bovine fasciolosis was significantly influenced by the breed of the animal (P < 0.05), with crossbred cattle being more heavily affected than local cattle; this aligns with Geja and Aliyi (2024). This discrepancy is widely believed to be driven by the superior genetic resistance of indigenous breeds to local parasitic pressures. Additionally, because crossbreeds require a larger volume of forage to sustain their production levels, their exposure window to contaminated pastures is significantly widened. When these high-yielding animals are reared under traditional, extensive husbandry systems, their likelihood of contracting the disease multiplies. Notably, these results run counter to those documented by Mathewos *et al*. (2023), and Mesfin *et al*. (2026), who found no significant correlation between animal breed and fasciolosis prevalence.

The current study indicated that the prevalence was significantly higher in cattle with poor body condition scores than good and medium body conditions. While this finding is aligned with Dereje and Surra (2018), El-Tahawy *et al*. (2017), Mohammed *et al*. (2018), Nigo *et al*. (2019), Khan *et al*. (2020), Shafi (2021), Mathewos *et al*. (2023), Geja and Aliyi (2024), Dubie *et al*. (2026), and Mesfin *et al*. (2026). The probable reason could be due to the fact that animals with poor body condition are usually less resistant and are consequently susceptible to various diseases including fasciolosis and due to reduced performance of the animals created by luck of essential nutrients and poor management by the animal owner. Similarly, prevalence of other concurrent infection (parasitic or non-parasites) might make the animal to have poor body condition.

Finally, the study revealed notable geographic variation in fasciolosis prevalence. Cattle originating from Entoto showed the infection rate, Kera, Beata, Family, suggesting that local environmental or management factors may exacerbate exposure to parasites. These differences highlight the importance of tailoring parasite control strategies to specific local conditions to maximize effectiveness.

## 5 Conclusion and recommendations

Fascioliasis is an economically devastating, neglected zoonotic disease of cattle caused by *Fasciola hepatica* and *Fasciola gigantica*. The present study showed that bovine fasciolosis is a prevalent disease in study areas affecting the well-being of the animals. The study confirmed that there are significant differences in the prevalence among age, animals' breed and physical state of study animals. Our observation generally suggested that bovine fasciolosis is an endemic condition in the study area and is an indication of the existence of favorable ecological conditions for survival, the multiplication and spread of the intermediate snail host and the parasite in that environment. It is important to consider probable risk factors for the disease's onset.

Based on the above conclusion the following recommendations are forwarded:

• Strategic use of anthelminthic should be performed to reduce pasture contamination with fluke eggs.
• Farmers should be aware of the transmission methods and control strategies of fasciolosis.
• Further epidemiological investigation on economic significance, and risk factor should be needed in the study area

### Animal subject

This study was conducted in accordance with the ARRIVE (Animal Research: Reporting of In Vivo Experiments) guidelines.

This study was approved by the Injibara University.

(Approval No. VET/LB/207.20/02/2026)

## CRediT authorship contribution statement

**Gashaw Molla Beza:** Conceptualization, Methodology, Validation, Investigation, Data curation, Writing – review & editing, Visualization, Supervision, Project administration. **Habtamu Addis:** Conceptualization, Methodology, Validation, Visualization, Supervision, Project administration, Funding acquisition. **Melaku Yismaw:** Conceptualization, Methodology, Software, Validation, Formal analysis, Investigation, Data curation, Writing – original draft, Writing – review & editing, Visualization.

## Declaration of competing interest

The authors declare no conflicts of interest**.**
